# Radiofrequency Ablation of the Splanchnic Nerve and Superior Hypogastric Plexus for Chronic Abdominal Pain Status Post-Abdominal Surgery

**DOI:** 10.7759/cureus.12189

**Published:** 2020-12-20

**Authors:** Nazir A Noor, Ivan Urits, Omar Viswanath, Lucien Alexandre, Alan D Kaye

**Affiliations:** 1 Anesthesiology and Critical Care, Mount Sinai Medical Center, Miami Beach, USA; 2 Anesthesiology, Louisiana State University Health Sciences Center, Shreveport, USA; 3 Pain Medicine, Southcoast Health, Boston, USA; 4 Anesthesiology, University of Arizona College of Medicine - Phoenix, Phoenix, USA; 5 Pain Medicine, Valley Pain Consultants, Phoenix, USA; 6 Anesthesiology, Creighton University School of Medicine, Omaha, USA; 7 Pain Management, Mount Sinai Medical Center, Miami Beach, USA

**Keywords:** interventional pain medicine, radiofrequency ablation, splanchnic nerve ablation, superior hypogastric plexus ablation, chronic abdominal pain

## Abstract

Gastrointestinal cancers, such as malignant carcinoid tumor and pancreatic cancer, are responsible for excruciating and debilitating abdominal pain. Too often, patients are placed on chronic high-dose opioids, but the pain remains poorly controlled. It is incumbent on the medical team to approach the patient’s debilitating pain in a thorough multi-modal fashion. Opioids may play an important role, but they make up only a portion of available invasive and noninvasive management.

We present a case of a patient who was serendipitously diagnosed with malignant carcinoid tumor after endoscopic polypectomy and Whipple procedure for pancreatic cancer. Her abdominal pain was refractory to opioid and non-opioid medications, and therefore we proposed radiofrequency ablation (RFA) of the splanchnic nerve and superior hypogastric plexus. This technique was preceded by a diagnostic block of these nerves. She experienced significant pain relief and an improved quality of life, and was able to stop all opioid medications.

The preferred approach to pain management is a multi-modal one. This includes physical therapy, pharmacological management, and minimally invasive procedures such as RFA. The medical team must consider all available pain management modalities to provide the patient with proper care of such debilitating pain as that described in our case presentation. A systematic approach is important, as demonstrated by our team by first performing diagnostic blocks of the superior hypogastric plexus and splanchnic nerve to test the likelihood of a successful RFA. Only after achieving favorable results, we decided to proceed with RFA treatment of those same nerves. Ultimately, our RFA technique provided significant pain relief for our patient and she did not require any opioid medications.

## Introduction

Pancreatic and intestinal cancers are notorious for causing excruciating abdominal pain. The patients are often prescribed high-dose opioids because non-opioid medications do not provide sufficient relief [1]. With the failure of traditional systemic pain management approaches for symptomatic relief, the next step would be to aim closer to the root cause of the pain with the help of radiofrequency ablations (RFAs) [2]. We present the case of a 61-year-old female who was able to experience significant relief from her chronic abdominal pain and stop taking her opioid medications after we performed an RFA of her superior hypogastric plexus and splanchnic nerve.

## Case presentation

Our case is that of a 61-year-old female with a prior history of serendipitously discovered malignant carcinoid tumor of the anterior duodenum status post-endoscopic polypectomy and pancreatic cancer status post-Whipple procedure. She continued to have chronic abdominal pain after these procedures and received an exploratory laparotomy and Roux-en-Y bypass with a partial gastrectomy. When she was first seen in the pain clinic, she complained of excruciating abdominal pain for six years. Oftentimes, her pain would be a 10/10 and that level of severity would remain for extended periods of time. She stated that this pain has affected many of her daily activities, including walking and sleeping. The pain had been refractory to ibuprofen, gabapentin, and duloxetine, among other non-opioid medications. She had also been placed on an array of opiates that included hydrocodone, tramadol, codeine, and fentanyl. When she was seen by our pain service, we began our plan of management conservatively. First, we provided trigger point injections in and around the abdominal scars left from her abdominal surgeries. This was followed by a trans-abdominis plane block. Neither of these provided relief. We then moved toward a diagnostic bilateral splanchnic nerve and superior hypogastric plexus block. She reported three days of 80% pain relief in her mid-epigastric region, and her lateral abdominal pain remained significantly improved. The next step in our treatment plan was an RFA of the bilateral splanchnic nerve and superior hypogastric plexus.

**Figure 1:**
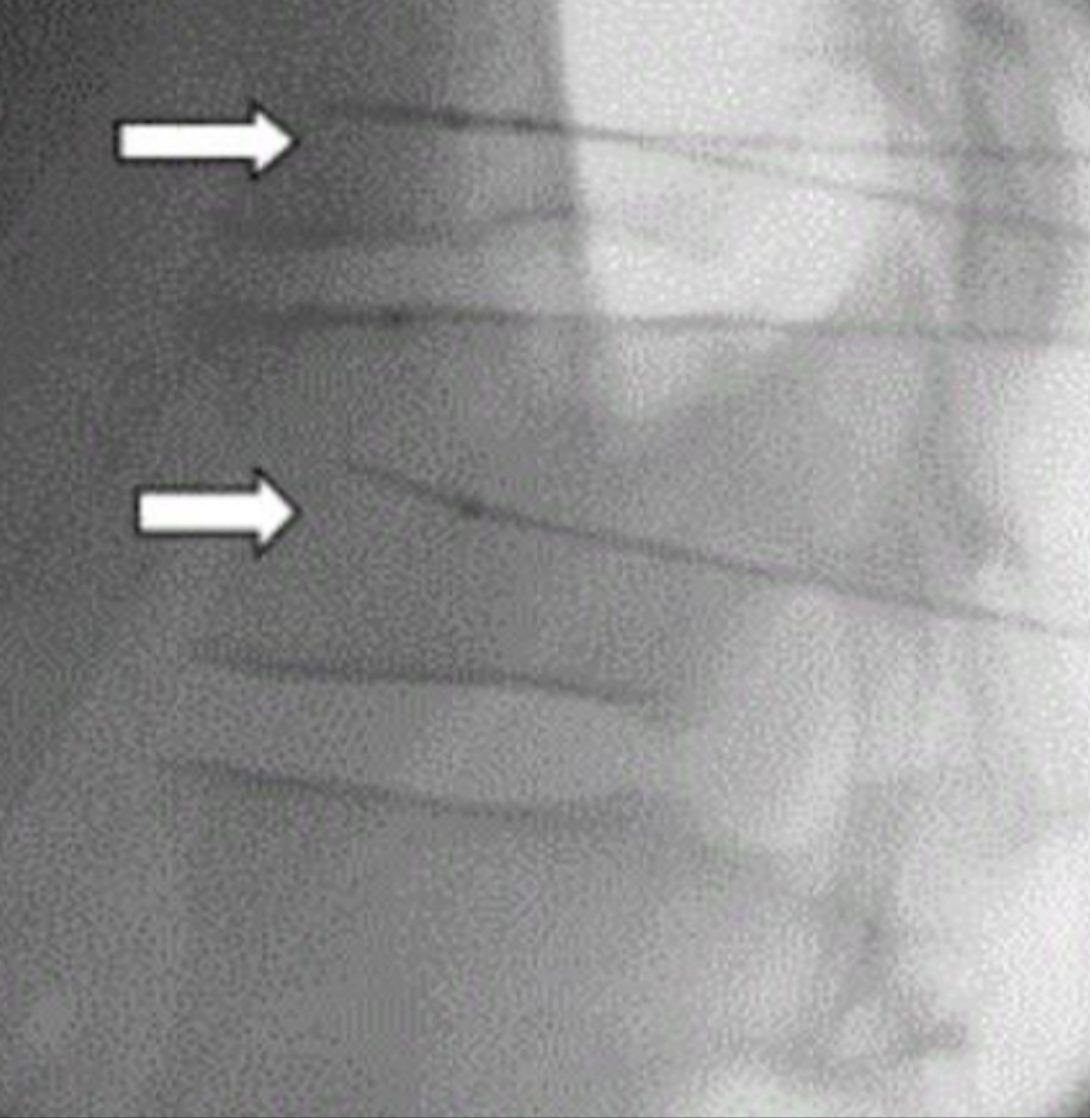
Lateral view of radiofrequency needles in the T10 and T11

The splanchnic nerve block has demonstrated effective pain control in cholangiocarcinoma patients [3]. Given this, we planned for a splanchnic nerve RFA for our patient. The patient was positioned prone. Anterior-posterior (AP) and lateral intermittent fluoroscopic views were used to identify the vertebral body and transverse processes of the right and left T11 vertebra. Prior to the radiofrequency (RF) needles being introduced and after negative aspiration, 2 mL of 2% lidocaine was injected to each of the four sites. Contrast was used under real-time fluoroscopy to rule out intravascular spread. Four 20-gauge 10-centimeter curved venom cannulas with active tip RF needles were advanced dorsal to the anterior vertebral body and caudad to the transverse process of the vertebra. AP and lateral radiographs were then taken to confirm proper needle placement, and no paresthesias were confirmed by the patient (Figure 1). The technique of needle placement and its confirmation is described in Kahan [3]. The stylet of each RF needle was removed and an RF probe was inserted through the cannula. The patient was then inquired about the sensory stimulation to an impedance between 300 and 800 ohms at 50 hertz to elicit abdominal discomfort. After both visual and patient-provided confirmation, RF denervation was performed at 60 degrees Celsius for 120 seconds for four cycles. After the RF denervation, 6 mL of 1% lidocaine was mixed with 2 mL of 40-mg/mL methylprednisolone, and 2 mL of this concoction was injected to each of the four sites of RFA.

**Figure 2:**
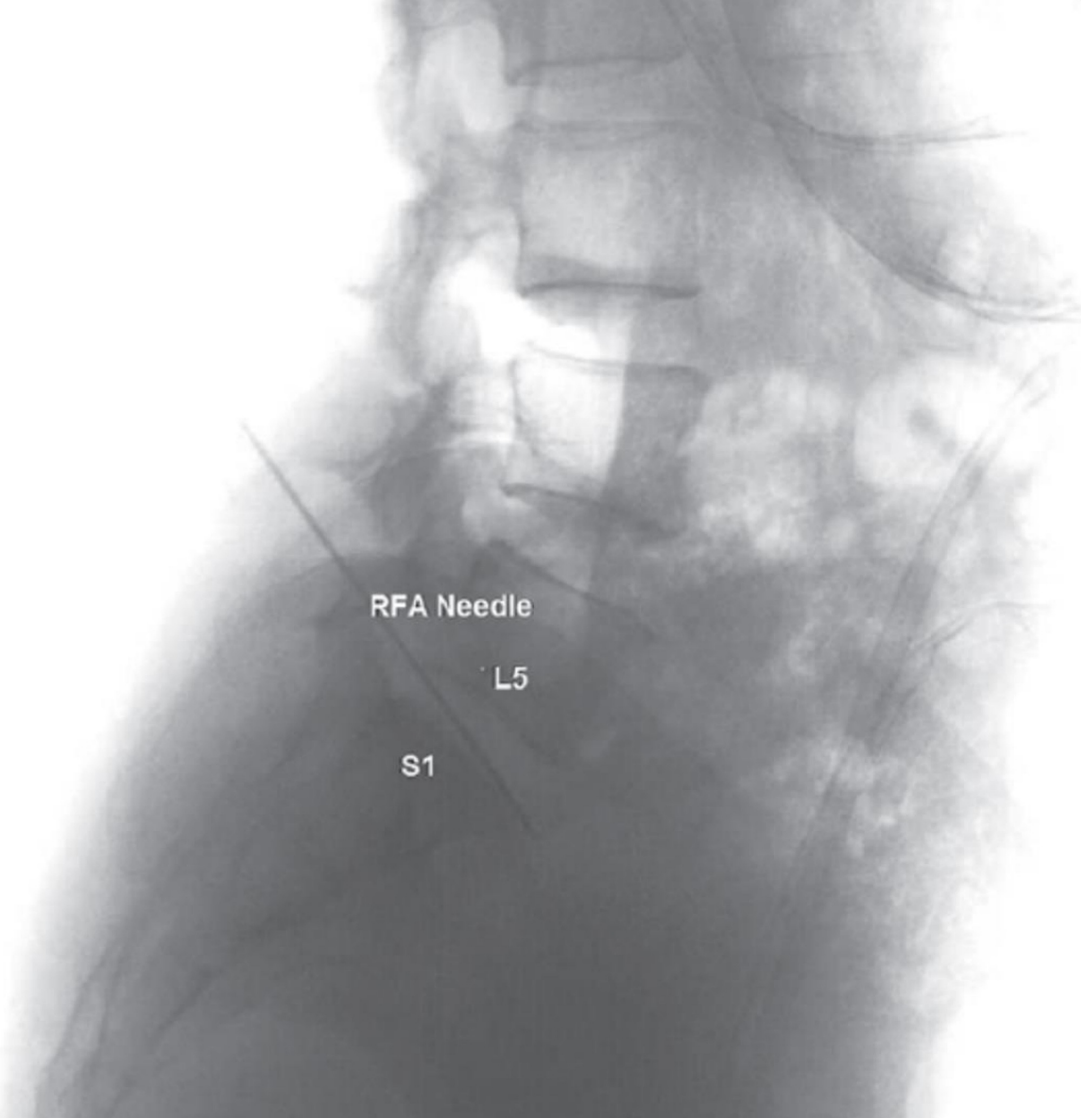
Lateral view of radiofrequency needles in L5-S1 RFA, radiofrequency ablation

**Figure 3:**
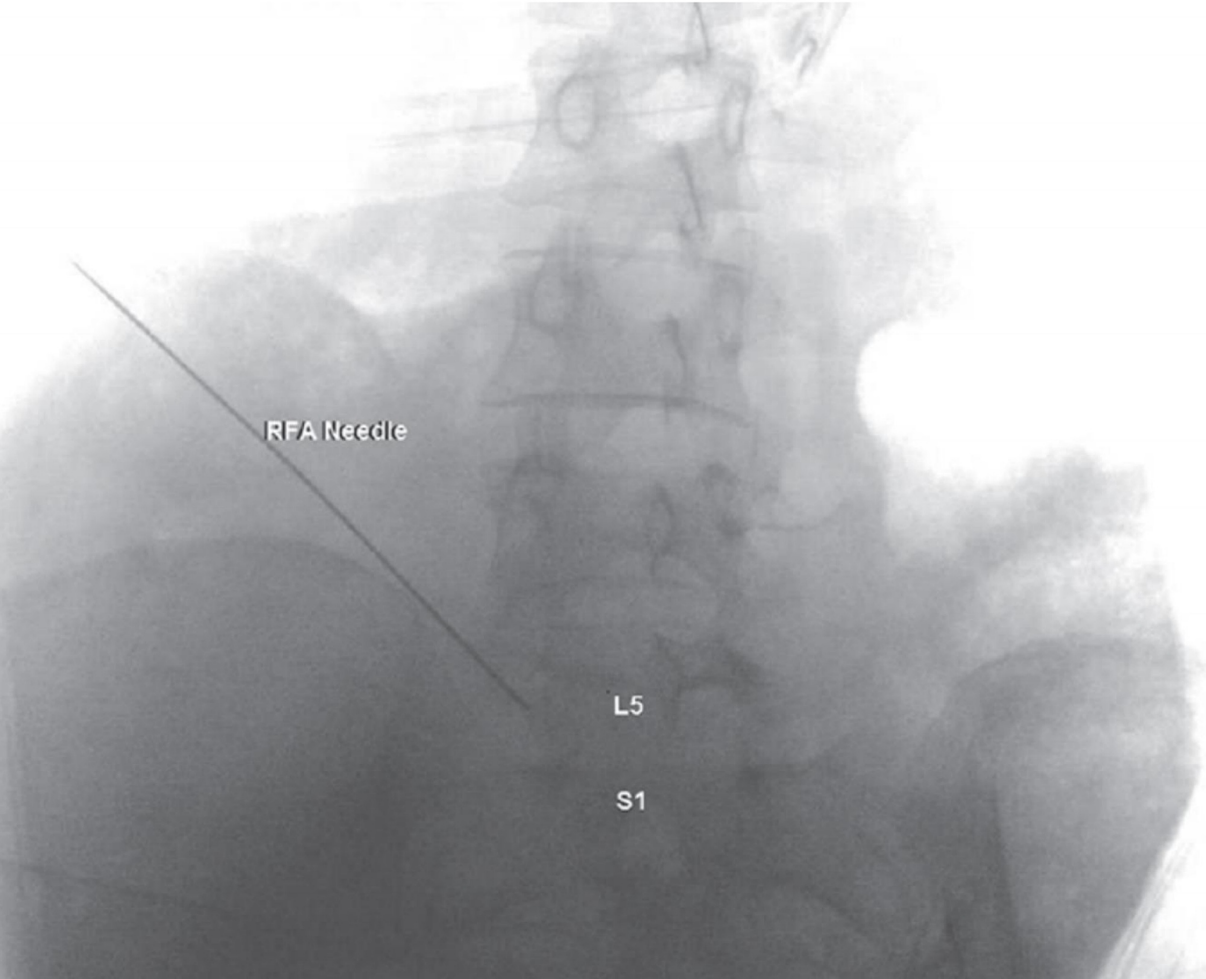
Anterior-posterior view of radiofrequency needle advancing in the L5-S1 intervertebral space RFA, radiofrequency ablation

For the superior hypogastric plexus RFA, the patient was again positioned prone. Fluoroscopic guidance in the oblique view was used to identify the right and left sides of the L5 vertebral body, such that the transverse process was partially superimposed along the lateral edge of the vertebral body. Once the target sites were under view and after negative aspiration, 2 mL of 2% lidocaine was injected to each of the four sites. Contrast was used under real-time fluoroscopy to rule out intravascular spread. Four 20-gauge 10-centimeter curved venom cannulas with active tip RF needles were advanced dorsal to the anterior vertebral body and caudad to the transverse process of the right and left L5 vertebral body. AP and lateral radiographs were then taken to confirm proper needle placement, and no paresthesias were confirmed by the patient (Figures 2, 3) [4]. The stylet of each RF needle was removed and an RF probe was inserted through the cannula. The patient was then inquired about the sensory stimulation to an impedance between 300 and 800 ohms at 50 hertz to elicit abdominal discomfort. After visual and patient-provided confirmation, RF denervation was performed at 60 degrees Celsius for 120 seconds for four cycles. After the RF denervation, 6 mL of 1% lidocaine was mixed with 2 mL of 40-mg/mL methylprednisolone, and 2 mL of this concoction was injected to each of the four sites of RFA. The procedure was well-tolerated by the patient. Upon follow-up after more than one month post-operatively, the patient reported 80% pain relief in both her mid-epigastric region and lower abdomen. Most importantly, she had stopped all opioid medications.

## Discussion

Patients may require high doses of opioids during the post-operative period after surgical removal or debulking of gastrointestinal cancers. The best approach to pain management is multimodal, and it may include some use of opioids, as is described by the World Health Organization's (WHO) stepwise pain management guidelines. In order to help provide opioid-sparing pain relief, it is important to utilize the WHO stepwise algorithm of pain management and not hesitate to utilize the available technique of RFA. Just like in our patient’s case, it is important not to skip steps as the practitioner moves down the algorithm of available treatments. We opted to test the likelihood of a successful RFA with splanchnic nerve and superior hypogastric plexus blocks. Only after these nerve blocks proved to provide clinically significant pain relief and an improved quality of life, per the patient, we decided to perform an RFA of the same nerves. Similar to Shah and Gulati’s study describing the successful treatment of chronic visceral abdominal pain with RFA and neurolysis, we were able to provide our patient with significant relief [1]. RFA of the splanchnic nerve was also used in the case reported by Kahan, where RFA of the splanchnic nerve was performed to treat cholangiocarcinoma pain [3]. Our case and others described in our presentation advocate for the use of RFA to provide long-lasting pain relief and allow the patient to minimize the use of opioids and other systemic pain medications. It is important to note that successful RFA of the splanchnic nerve and superior hypogastric plexus, like all other procedures, is patient-specific. The frequency of the procedure is usually much less than a nerve block, making it a much more cost-effective and desirable approach to the management of chronic abdominal pain. There is still room for improvement, such as better localization of the nerves, the temperature used, or the duration of time used for the ablation since our method provided 80% relief.

## Conclusions

RFA of the splanchnic nerve and superior hypogastric plexus is an effective option for chronic abdominal pain refractory to other pain management models. Our case demonstrated its opioid-sparing and significant pain-relieving benefits. This case further advocates for a proper approach to multi-modal pain management that is described by the WHO pain management guidelines. Further research involving large clinical trials will provide us with a better understanding of the technique and efficacy of such procedures for chronic pain management. With a better understanding, we can take further steps toward utilizing available treatment options that will help reduce and even avoid the use of opioids.
